# Dynamic Telomerase Gene Suppression via Network Effects of GSK3 Inhibition

**DOI:** 10.1371/journal.pone.0006459

**Published:** 2009-07-31

**Authors:** Alan E. Bilsland, Stacey Hoare, Katrina Stevenson, Jane Plumb, Natividad Gomez-Roman, Claire Cairney, Sharon Burns, Kyle Lafferty-Whyte, Jon Roffey, Tim Hammonds, W. Nicol Keith

**Affiliations:** 1 Centre for Oncology and Applied Pharmacology, University of Glasgow, Cancer Research UK Beatson Laboratories, Garscube Estate, Bearsden, Glasgow, United Kingdom; 2 Cancer Research Technology Ltd., Wolfson Institute for Biomedical Research, The Cruciform Building, London, United Kingdom; University of Arkansas for Medical Sciences, United States of America

## Abstract

**Background:**

Telomerase controls telomere homeostasis and cell immortality and is a promising anti-cancer target, but few small molecule telomerase inhibitors have been developed. Reactivated transcription of the catalytic subunit *hTERT* in cancer cells controls telomerase expression. Better understanding of upstream pathways is critical for effective anti-telomerase therapeutics and may reveal new targets to inhibit *hTERT* expression.

**Methodology/Principal Findings:**

In a focused promoter screen, several GSK3 inhibitors suppressed *hTERT* reporter activity. GSK3 inhibition using 6-bromoindirubin-3′-oxime suppressed *hTERT* expression, telomerase activity and telomere length in several cancer cell lines and growth and *hTERT* expression in ovarian cancer xenografts. Microarray analysis, network modelling and oligonucleotide binding assays suggested that multiple transcription factors were affected. Extensive remodelling involving Sp1, STAT3, c-Myc, NFκB, and p53 occurred at the endogenous *hTERT* promoter. RNAi screening of the *hTERT* promoter revealed multiple kinase genes which affect the *hTERT* promoter, potentially acting through these factors. Prolonged inhibitor treatments caused dynamic expression both of *hTERT* and of c-Jun, p53, STAT3, AR and c-Myc.

**Conclusions/Significance:**

Our results indicate that GSK3 activates *hTERT* expression in cancer cells and contributes to telomere length homeostasis. GSK3 inhibition is a clinical strategy for several chronic diseases. These results imply that it may also be useful in cancer therapy. However, the complex network effects we show here have implications for either setting.

## Introduction

Telomerase is a ribonucleoprotein reverse transcriptase which counteracts telomere attrition in dividing cells by synthesising telomere DNA [1]. Telomerase activity requires the catalytic subunit hTERT and the RNA subunit *hTERC*, which contains the template sequence for reverse transcription. Both gene products are over-expressed in cancer cells relative to somatic cells and in most human cancers. Telomere homeostasis is essential for cell immortalisation and telomerase is an attractive anti-cancer target [2].

Telomerase expression in cancer cells is dependent on aberrant *hTERC* and *hTERT* transcription, resulting from multiple events including altered signalling and changes in the promoter chromatin environments relative to normal cells [3]. However, the cloned promoters also have cancer cell specific activity, leading many groups to develop telomerase-specific gene therapy models [4]. Several transcription factors affecting each gene promoter are known. The *hTERT* promoter, for example, is regulated by multiple factors including Myc, Mad, Sp1, STATs, E2F and p53, among others [5].

Current clinical trials of telomerase therapeutics include several immunotherapeutics, an oncolytic adenovirus, and GRN163L, a modified oligonucleotide telomerase inhibitor [2], [5], [6]. Targeting telomerase transcription using signal transduction inhibitors may also hold value [2], [7]. However, signalling events upstream of the telomerase genes remain poorly understood and in most studies in which signal transduction inhibitors have been found to affect expression of telomerase genes, long term treatments to examine effects on telomere length and telomere dependent senescence have not been performed. In this study, we tested whether focused cell-based screening using well-defined kinase inhibitors could provide a platform to identify new telomerase regulatory pathways and candidate targets for pharmacological intervention.

We show that glycogen synthase kinase 3 (GSK3) activates *hTERT* transcription and characterise the pathway upstream of *hTERT*. GSK3 inhibition reduced *hTERT* promoter activity, expression, telomerase activity and telomere lengths in several cell lines and suppressed tumour growth and *hTERT* expression in a xenograft model. Therefore, GSK3 inhibition may be an appropriate anti-cancer strategy. Prolonged GSK3 inhibition in A2780 cells profoundly reduced telomere lengths; interestingly however, *hTERT* expression was not stably suppressed but showed dynamic oscillation.

GSK3α and β isoforms, which are both targets of GSK3 inhibitors, variously regulate diverse cellular processes including survival and apoptosis, energy metabolism, cell fate specification and stem cell self renewal through phosphorylation of multiple substrates in several distinct pathways including Wnt and insulin signalling [8], [9]. We present a network model of *hTERT* activation and show that GSK3 inhibition affects multiple transcription factors converging on *hTERT*. Interestingly, expression levels of several transcription factors were also dynamically regulated under prolonged GSK3 inhibitor treatments, suggesting that GSK3 may control steady state behaviour of the network. A whole-kinome RNAi screen of the *hTERT* promoter is interpreted using this model to predict rational combinatorial targets to enhance anti-telomerase effects of GSK3 inhibitors.

## Results

### GSK3 activates the *hTERT* promoter

**Figure 1:**
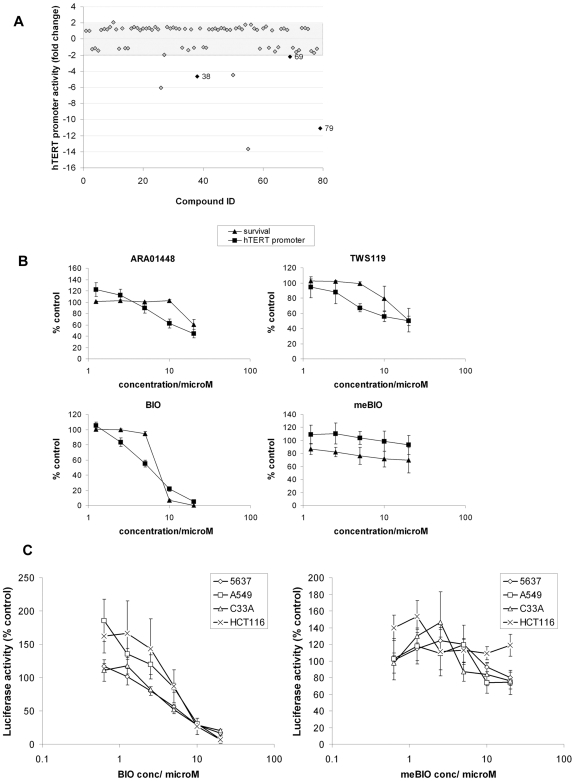
GSK3 inhibitors suppress the *hTERT* promoter. (A) Kinase inhibitor screen: A2780 cells were transfected with *hTERT*-luciferase reporter. 32 h later cells were treated for 16 h with DMSO or 10 µM kinase inhibitors prior to luciferase assay. Hits are shown outside the shaded area. 38: Ro-31-8220; 69: indirubin-3′-monoxime; 79: kenpaullone. Mean of 3 experiments. (B) *hTERT* promoter inhibition and toxicity of GSK3 inhibitors. A2780 cells were transfected with *hTERT*-luciferase reporter. 32 h later cells were treated for 16 h with compounds at 20 µM, 10 µM, 5 µM, 2.5 µM and 1.25 µM or DMSO for 16 h prior to luciferase assay. Parallel MTT assays of compound toxicity were performed. Mean±SEM of 3 experiments. (C) BIO suppresses the *hTERT* promoter in multiple cell lines. Cells were transfected with *hTERT*-luciferase reporter. 32 h later cells were treated for 16 h with inhibitor titrations as in (B) prior to luciferase assay. Mean±SEM of 3 experiments.

In a focused screen of 79 well characterised kinase inhibitors, A2780 cells were transfected with *hTERT* reporter construct and 32 h post transfection were exposed to 10 µM each inhibitor for 16 h. Six compounds suppressed promoter activity by at least 2-fold (figure 1A). Compounds 38 (Ro-31-8220, bis indole maleimide family; 4.6-fold), 69 (indirubin-3′-monoxime, indirubin core; 2.2-fold) and 79 (kenpaullone, indolo benazepinone core; 11.1-fold) are all reported to inhibit GSK3 [10]. The other hit compounds were: 26, tyrphostin AG 1295 (inhibitor of PDGFR [11]); 50, 5-iodotubercidin (inhibitor of adenosine kinase [12]); and 55, SU4312 (inhibitor of PDGFR and FGFR [13]).

To extend the observation that diverse GSK3 inhibitors suppress *hTERT* promoter activity, we performed *hTERT* reporter assays and parallel MTT assays, titrating the selective inhibitors AR-A014418 (benzyl-thiazolyl urea substructure), TWS119 (pyrollopyrimidine core), and 6-bromoindirubin-3′-oxime (BIO, indirubin core) alongside the GSK3-inactive BIO derivative 1-methyl-BIO (MeBIO) [10]. Structures of all GSK3 inhibitors used in this study are available in supporting figure S1. All supporting figure and file legends are given in supporting file S4. Active inhibitors of different chemotypes, but not MeBIO, suppressed *hTERT* promoter activity at sub-toxic concentrations (figure 1B). At optimal doses of 10 µM (AR-A01448) and 5 µM (TWS119 and BIO) promoter activities were 62%, 67% and 55% of control. BIO and MeBIO were also titrated against the *hTERT* reporter in 5637, C33A, A549 and HCT116 cells (figure 1C). BIO, but not MeBIO, suppressed the *hTERT* promoter in all cells with IC50s in the range 5.4 µM-8.2 µM. Similarly, BIO, but not MeBIO activated Topflash reporter activity in all cells, indicative of specific GSK3β inhibition (supporting figure S2).

**Figure 2:**
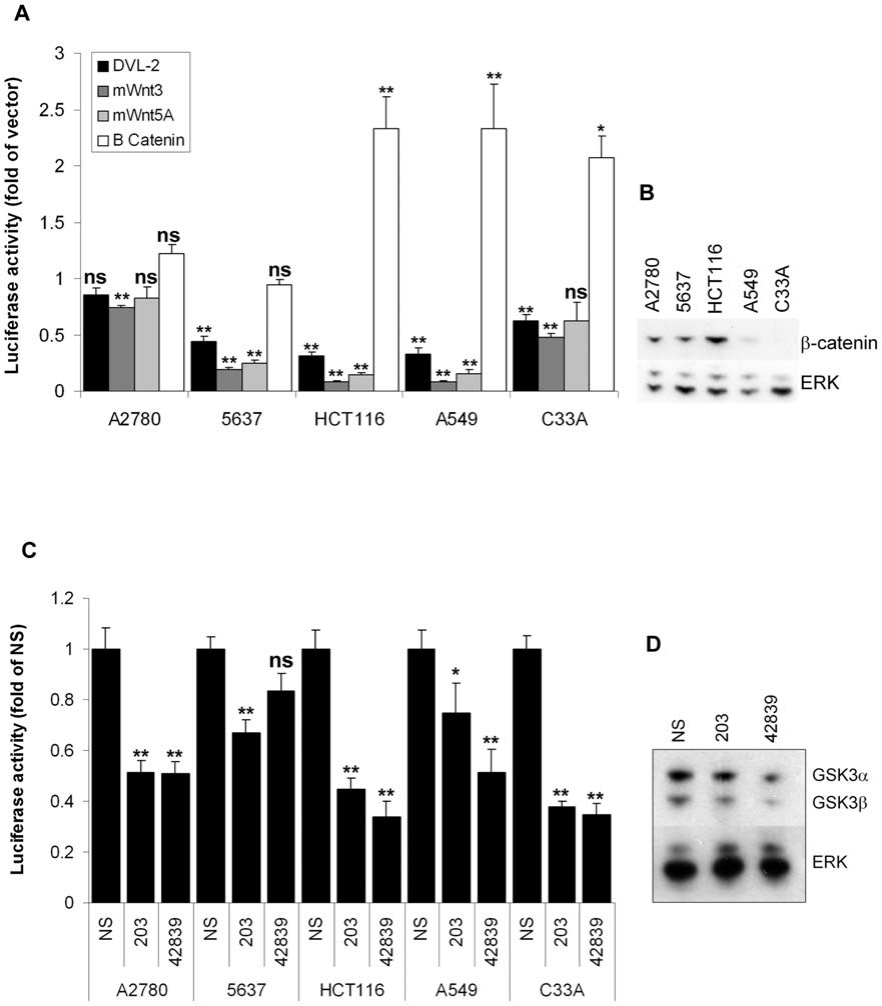
GSK3β activates the *hTERT* promoter. (A) Wnt signalling inhibits the *hTERT* promoter. A2780 cells were transfected with *hTERT*-luciferase and the CMV expression vectors shown. 48 h later reporter activities were determined relative to empty vector. Mean±SEM of 3 experiments (ns: not significant; *: p<0.5; **: p<0.01). (B) Basal β-catenin expression in 20 µg protein samples was analysed by western blotting. The experiment was performed twice. Representative blots are shown. (C) GSK3β RNAi inhibits the *hTERT* promoter. A2780 cells were transfected with *hTERT*-luciferase and 50 nM non-specific (NS) siRNA or GSK3β specific siRNAs 203 or 42839. 48 h later reporter activities were determined. Mean±SEM of 3 experiments (ns: not significant; *: p<0.5; **: p<0.01). (D) Knockdown of GSK3β by siRNA-203 and -42839. A2780 were transfected with 50 nM siRNA and harvested after 48 h. 20 µg protein samples were analysed by western blotting. The experiment was performed twice. Representative blots are shown.

To further characterise the effect of GSK3 on the *hTERT* reporter we over-expressed mWnt3, mWnt5A or human DVL2 in each cell line, which all inhibit GSK3β in Wnt signalling. Additionally, we examined the effect of β-catenin over-expression. Both Wnts and DVL2 reduced *hTERT* promoter activity in 5637, HCT116 and A549. Wnt3 had the strongest effect and DVL2 the weakest (figure 2A). Only mWnt3 had a significant effect in A2780 and the effect of mWnt5A was not significant in C33A. Promoter repression was not correlated with basal β-catenin expression (figure 2B). For example, despite low expression in A549 and constitutively stable expression in HCT116, each construct had similar effects, reducing promoter activity to 32% of control (mWnt3), 8% (mWnt5A) and 14% (DVL2) in HCT116 and 33%, 8%, and 15%, respectively, in A549. Interestingly, wild type β-catenin induced promoter activity in HCT116, A549 and C33A. Supporting figure S3 shows the effect of each construct on Topflash reporter activity in each cell line. β-catenin and DVL2 generally resulted in the strongest increase of Topflash activity, whereas mWnt5A had a weaker effect. Interestingly, mWnt3 mildly decreased Topflash activity in some cells. The effect of each transfection on canonical Wnt signalling presumably depends on the specific status of the pathway in each cell type.

To confirm that GSK3β regulates the *hTERT* promoter, we co-transfected each cell line with reporter and with 50 nM GSK3β-specific siRNA (two GSK3β-specific siRNA were tested–siRNA-203 and siRNA-42839). Both siRNAs tested reduced promoter activity in all cells relative to non-specific control (Figure 2C). All effects were significant except siRNA-42839 in 5637 cells. Promoter activities ranged between 38%–75% of control for siRNA-203 and 34%–83% for siRNA-42839 at 48 h post-transfection. Both specific siRNAs produced a GSK3β knockdown in A2780 at 50 nM (figure 2D). SiRNA-42839 produced a greater knockdown than siRNA203 and, interestingly, also resulted in some knockdown of GSK3α. The GSK3α transcript is highly homologous with that of GSK3β in the 42839 target site, sharing 16/19 nucleotides. It is possible that GSK3α knockdown might result from target sequence homology. However, given the high nucleotide specificity of the RNAi mechanism, it is perhaps more plausible that GSK3β affects GSK3α expression. Together these data confirm that GSK3 activates the cloned *hTERT* promoter in multiple cell lines.

### Functional specificity of BIO

**Figure 3:**
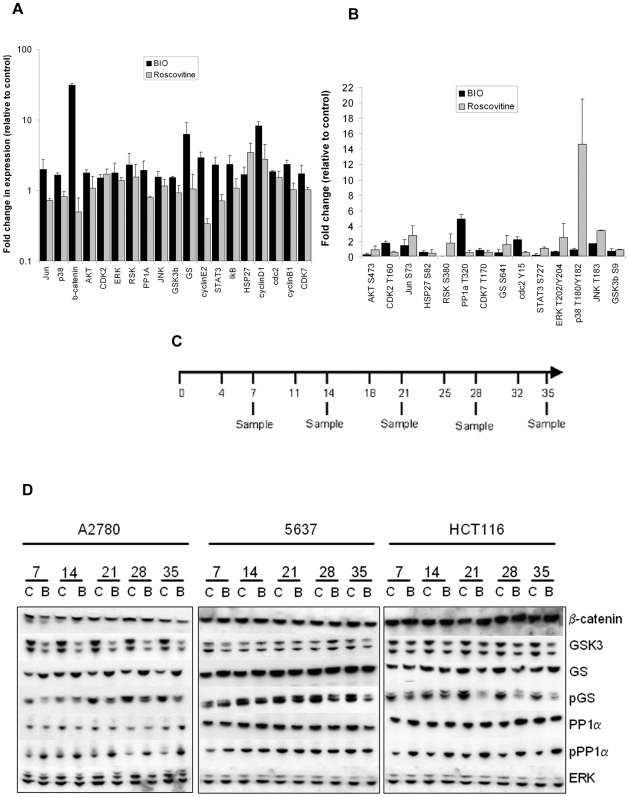
Specificity of BIO. A2780 cells were treated for 16 h with 5 µM BIO, 10 µM roscovitine, or DMSO. 1 mg protein samples were analysed by multiplex western blotting to detect (A) expression and (B) phosphorylation of indicated proteins. Band intensity changes were assessed by densitometry relative to DMSO. Phosphospecific bands are normalised with expression. Mean±SEM of 2 experiments. (C) BIO schedule in 5 week treatments. Cells were treated twice weekly on days 1 and 4 with DMSO or BIO (A2780, 2.5 µM; HCT116, 1 µM; 5637, 500 nM). BIO was not removed between treatments. Counting and harvesting was on treatment day 1 of each week. (D) GSK3 inhibition over 5 week BIO treatment. 20 µg protein samples from each time point were analysed by western blotting (C, Control; B, BIO). Two independent treatments were analysed. Representative blots are shown.

Specificity of the GSK3 inhibitor BIO was assessed by phospho-specific multiplex western blotting. A2780 were treated with DMSO, 5 µM BIO, or 10 µM roscovitine to control for inhibition of possible off-target kinases CDK1, 2, and 5 [10]. Protein samples from treated cells were separated by SDS-PAGE using single-well 10% Bis-Tris minigels and blotted onto PVDF filters. 28 individual lanes were isolated on the filters using a miniblotter dual apparatus. To provide a detailed view of effects on GSK3 signalling, each lane was probed with an individual antibody or antibody cocktail to quantify expression (figure 3A) and phosphorylation (figure 3B) of multiple proteins involved in GSK3 signalling and other pathways. Intensity change of quantifiable bands relative to control was assessed by densitometry. Note the log scale in figure 3A.

BIO treatment strongly induced β-catenin (30.9-fold), cyclin D1 (8.2-fold) and glycogen synthase (GS) (6.3-fold), which are all destabilised by GSK3 [14]–[16]. Expression of cyclins B1 and E2, p90-RSK, STAT3, c-Jun and IκB were also increased by around 2–3 fold each. It has previously been reported that c-Jun and IkB stability are also regulated by GSK3 [17], [18]. Roscovitine also mildly induced cyclin D1 (2.7-fold) but, in contrast with BIO, decreased β-catenin (49% of control) and cyclin E2 (34%). As previously reported, roscovitine induced MAPK phosphorylation [19], while BIO blocked phosphorylation on AKT S473 (29% of control), p90-RSK S380 (12%), STAT3 S727 (26%), and GS (56%) and induced PP1α T320 phosphorylation (4.9-fold). Thus, the effects of BIO are consistent with GSK3 inhibition and largely non-overlapping with those of a pan-CDK inhibitor.

### BIO inhibits endogenous telomerase

To determine whether GSK3 inhibition suppresses endogenous telomerase, 5637, HCT116 and A2780 cells were cultured continuously in log phase with BIO or DMSO given twice-weekly for five weeks. Drug was not removed between treatments. Cells were counted weekly and at each time point individual cell pellets were taken from both control and treated cells for telomere length analysis, *hTERT* expression analysis by RT-QPCR, microarray analysis, western blotting of GSK3 inhibition markers, and for TRAP analysis of telomerase activity (figure 3C). At the time of sampling, drug had not been replenished for 3 days. BIO concentrations used in these experiments were 2.5 µM (A2780), 1 µM (HCT116) and 500 nM (5637).

Western blotting was performed to detect levels of β-catenin, GSK3 and expression and phosphorylation of GS and PP1α at each sampling time point (figure 3D). β-catenin is constitutively stable in HCT116 but was also unaffected in the other cell lines, presumably indicating rapid return to basal levels as the effects of BIO diminish between treatment and sampling. However, GS S641 phosphorylation decreased progressively in all BIO treated cells and its expression was also increased throughout in A2780. Therefore, GSK3 was inhibited in all cell lines. Additionally, increased PP1α T320 phosphorylation and reduced GSK3α and β expression were detected at all times in A2780 and HCT116.

**Figure 4:**
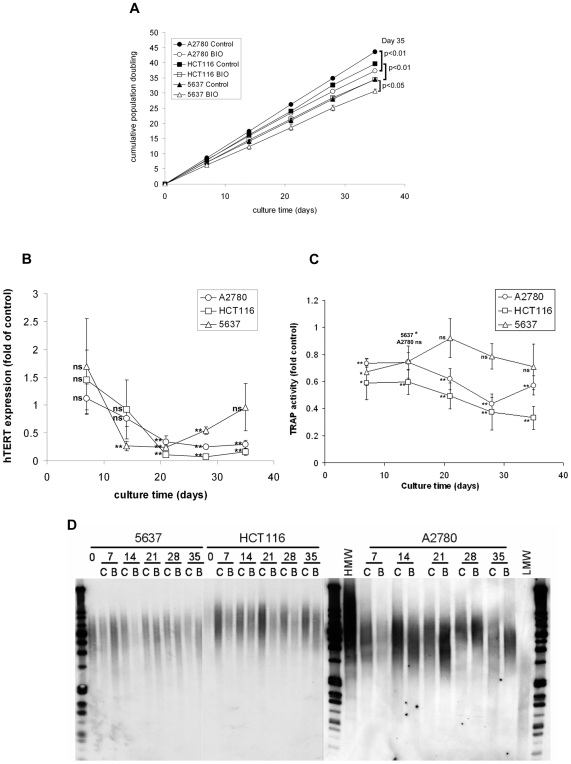
BIO inhibits telomerase. (A) 5 week cell growth curves under BIO treatment. Cells were counted weekly to determine cumulative population doublings (PD). Mean±SEM of three experiments. (B) BIO represses *hTERT* expression. Control and treated samples from each time point were analysed by Q-RTPCR for *hTERT* expression normalised to *RPS15*. Mean±SEM of *hTERT* expression in BIO treated cells relative to control from three experiments (ns: not significant; **: p<0.01). (C) BIO represses telomerase activity. Telomerase activity was determined by Q-PCR TRAP analysis in control or BIO treated cells. Mean±SEM of treated cells relative to controls from three experiments (ns: not significant; *: p<0.05; **: p<0.01). (D) BIO shortens telomeres. 1 µg genomic DNA from control or treated cells was digested with HinfI/RsaI and southern blotted with DIG-labelled telomere detection probe (C, Control; B, BIO; HMW and LMW, high and low molecular weight markers). Two independent treatments were analysed. Representative blots are shown.

Prolonged telomerase inhibition is predicted to result in growth plateau by analysis of cumulative population doublings (PD), but this was not observed over five week BIO treatment, though treated cells grew more slowly than controls (Figure 4A). Average growth rates were around 7 PD/week (control) and 6 PD/week (BIO) for 5637 and 8 PD/week (control) and 7 PD/week (BIO) for HCT116. However, A2780 growth rates decreased under BIO treatment. Controls grew steadily at approximately 8.5 PD/week, whereas BIO treated cells had an initial rate of 8 PD/week, slowing to <7 PD by day 35.

RT-QPCR analysis confirmed that BIO suppressed *hTERT* expression in all three cell lines (figure 4B). In A2780, *hTERT* expression under BIO treatment was reduced to 25% of control on day 28. Expression in treated HCT116 was 7% of control on day 28 and in 5637 was 24% of control on day 21. Analysis of *hTERT* splice variants in A2780 cells revealed selective repression of the full length transcript (supporting figure S4). We observed that *hTERT* expression in BIO treated 5637 returned to control levels on day 35. This observation is expanded below.

We next performed QPCR-TRAP analysis using the TRAPeze XL kit to determine telomerase activity in control and treated samples. Cell pellets from each time point were lysed in CHAPS buffer and protein samples incubated with reaction mix containing TS and RP primers in addition to the control K2 primer. Each assay included no-telomerase, no-Taq, and heat-treated controls. QPCR detection of fluorescein labelled RP product confirmed that telomerase activity was reduced by BIO (figure 4C). TRAP activity was reduced in all BIO treated cells by day 7 (A2780, 74% of control; HCT116, 59%; 5637, 67%) and generally diminished over the treatment, reaching 44% of control in A2780 on day 28, then increasing slightly to 57% on day 35. In 5637, TRAP activity rose on days 14 and 21, approaching control activity before recommencing a downward trend to reach 71% by day 35, though this reduction relative to control was not statistically significant. HCT116 TRAP activity decreased continuously to 33% of control levels by day 35.

Telomere lengths were reduced by BIO in all cell lines as determined by telomere restriction fragment (TRF) analysis (figure 4D). Genomic DNA was extracted from control and treated cell pellets and digested with HinfI/RsaI. Digestion products were separated by electrophoresis and analysed by Southern blotting using a DIG-labelled telomere sequence probe to determine telomere length range. The decreases were small and were most evident at later time points, consistent with the short treatment period. In HCT116 we also observed reduction of the overall signal in treated samples. Therefore, GSK3 inhibition suppresses *hTERT* expression, telomerase activity and decreases telomere lengths in several cancer cell lines.

### Network model of *hTERT* regulation

To characterise the mechanism of *hTERT* repression, we performed microarray expression analysis using cDNA from day 21 control or BIO treated A2780. Three independent treatments were analysed in duplicate. Mean intensity of 1048 differentially expressed transcript IDs changed by >5-fold, p<0.01 between control and treated cells across all repeats. Raw Agilent ID list with fold intensity changes of the differentially expressed genes used for modelling are available in supporting file S2. The full MIAME compliant array data have been deposited for public access in the Gene Expression Omnibus. The profile included multiple transcriptional targets of Wnt signalling, such as uPAR, EphB, Runx2, stromelysin, Irx3, Pitx2, Islet1, Tcf-1, LEF1, dickkopf-4, axin-2, Wnt5B and Wnt11, consistent with ongoing inhibition of GSK3 on treatment day 21.

Network modelling was performed on the profile using MetaCore from Genego Inc [20]. 622 unique database objects were recognised (note that several tags may correspond to a single gene). We first identified networks centred on individual high-degree transcription factor neighbours of differentially expressed genes using the transcription-regulation algorithm (supporting figure S5). All 144 unique genes from the ten highest scoring networks were combined in an enriched list and a best-fit transcriptional network was generated using the auto-expand algorithm. Network size was optimised to include all differentially expressed genes from the enriched list (23.2% of all input genes) (supporting figure S6).

**Figure 5:**
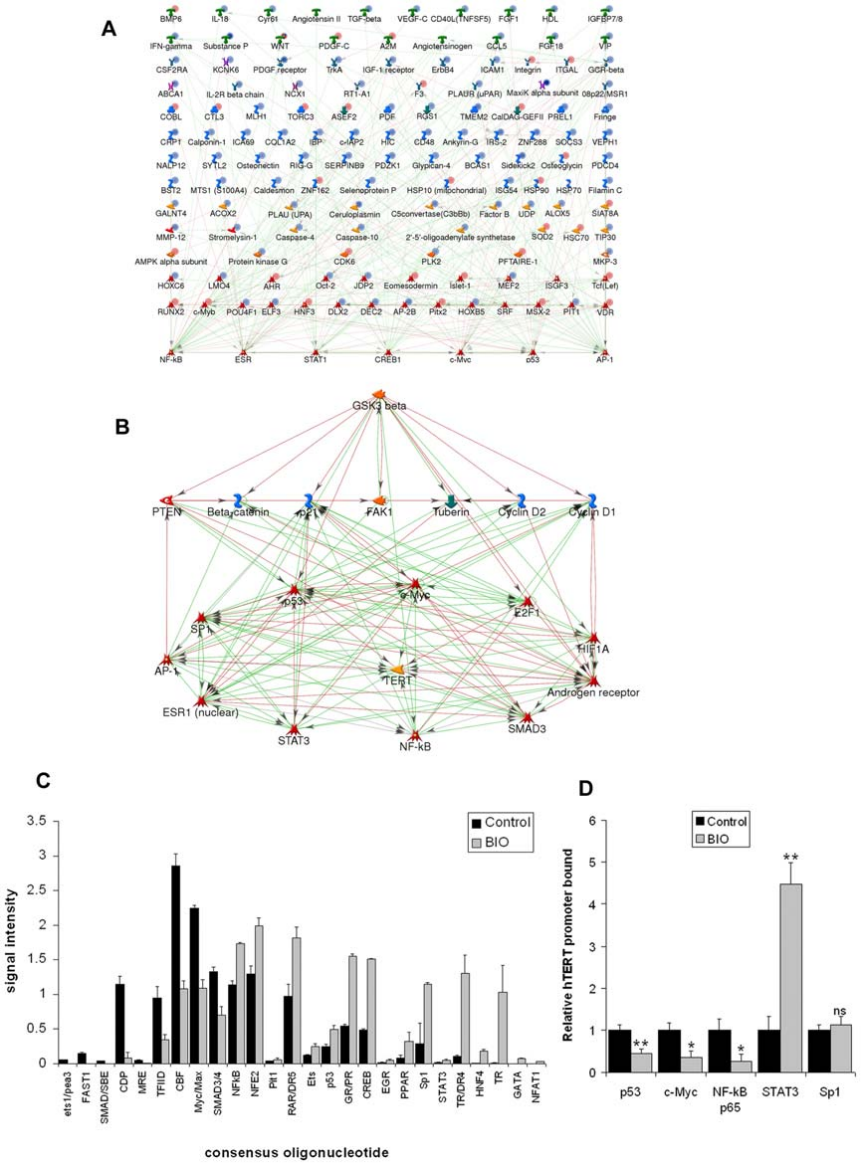
Network modelling and validation. (A) Transcriptional network of GSK3 inhibition. Differentially expressed genes from day 21 BIO treated cells were identified using Agilent whole genome expression arrays (n = 3; mean fold change>5; p<0.01). High degree transcription factor neighbours of differentially expressed genes were identified by network analysis in MetaCore. Blue circles: down-regulated in BIO treated cells; red circles: up-regulated. Shading intensity indicates fold change (minimum 5-fold). Green arrows: activation; red: inhibition (reaction mechanisms not shown). (B) Candidate network linking GSK3 to *hTERT*. Shortest paths linking transcription factors from (A) and GSK3 were identified in MetaCore. Green arrows: activation; red: inhibition; reaction mechanisms not shown. (C) Multiplex consensus oligonucleotide binding in A2780 nuclear extracts. 16 h DMSO or BIO treated cell nuclear extracts were incubated with labelled consensus oligonucleotide probe mix prior to column purification and hybridisation of eluted bound probes to oligonucleotide array. Mean±SEM of duplicate spots. (D) *hTERT* promoter remodelling. A2780 were treated for 16 h with DMSO or 5 µM BIO prior to Chromatin IP (ChIP) with antibodies shown and QPCR detection of the *hTERT* promoter. Mean±SEM of three experiments (ns: not significant; *: p<0.05; **: p<0.01).

The analysis returned a network involving NFκB, ESR1, STAT1, CREB1, c-Myc, p53 and AP-1 (figure 5A; blue and red circles adjacent to network object icons represent fold intensity change with values given in supporting file S3). Thus, altered activity of these transcription factors may significantly contribute to the observed BIO treatment profile. We therefore determined shortest paths between GSK3 and these high-degree nodes using the analyse-network algorithm (figure 5B). Interestingly, *hTERT* is returned by this analysis as a high probability component of the final network, defining a candidate network linking GSK3 and telomerase. For clarity, only network objects downstream of GSK3 or upstream of *hTERT* are shown. The analysis suggests that multiple transcription factors may participate in regulation of *hTERT* by GSK3, including some or all of Sp1, E2F1, SMAD3, STAT3, HIF-1α, Androgen Receptor (AR), p53, c-Myc, ESR1, AP-1 and NFκB. All are reported to regulate *hTERT* or its cloned promoter [5]. GSK3 inhibition may affect their activities both directly and through several effectors.

### Network validation

To validate the model, we first performed a multiplex consensus oligonucleotide binding assay using the protein/DNA array I kit from Panomics. 56 biotin-labelled double stranded consensus transcription factor binding site probes were mixed with nuclear extracts of A2780 treated for 16 h with DMSO or 5 µM BIO. DNA-Protein complexes were bound to spin columns and unbound probe washed off. Bound probes were then eluted and hybridised to an unlabelled membrane array containing spots of each consensus sequence. Hybridisation of labelled probes to the membrane, indicative of binding to the nuclear extract, was detected with strptavidin-HRP. Figure 5C shows densitometry of quantifiable spots showing >1.5-fold intensity change in BIO treated cells.

Signals for c-Myc, and Smad3/4 binding sequences were reduced by 2.1-fold and 1.9-fold, respectively. Increased signal was detected for NFκB (1.5-fold), p53 (2-fold), Sp1 (3.9-fold) and STAT3 (3.9-fold) consensus sequences. HIF-1α was not represented on the array and we did not detect altered binding to sequences for ESR1, AR, AP-1, or E2F1. However, longer treatments may also affect these factors. Several other consensus sequences also showed altered binding activity and their cognate transcription factors may also participate in the overall effect of GSK3 inhibition.

To validate the model with respect to *hTERT*, we performed chromatin immunoprecipitation (ChIP) of Sp1, STAT3, p53, c-Myc, and NFκB p65 in 16 h BIO or DMSO treated A2780. QPCR detection of the *hTERT* promoter in precipitates revealed widespread promoter remodelling, affecting both activators and repressors (Figure 5D). Detectable immunoreactive epitopes of c-Myc, p65 and p53 were reduced 2.9-fold, 4-fold and 2-fold respectively, while that of STAT3 was increased 4.5-fold, confirming that GSK3 inhibition rapidly affects the *hTERT* promoter environment involving direct regulation by at least four factors suggested by network analysis. Sp1 binding to the *hTERT* promoter was unaffected by 16 h BIO treatment. Interestingly, *hTERT* expression in 16 h BIO treated A2780 was not significantly repressed and was even slightly increased, suggesting that promoter remodelling may proceed dynamically in longer treatments (supporting figure S7).

### BIO inhibits *hTERT* expression and tumour growth in xenografts

**Figure 6:**
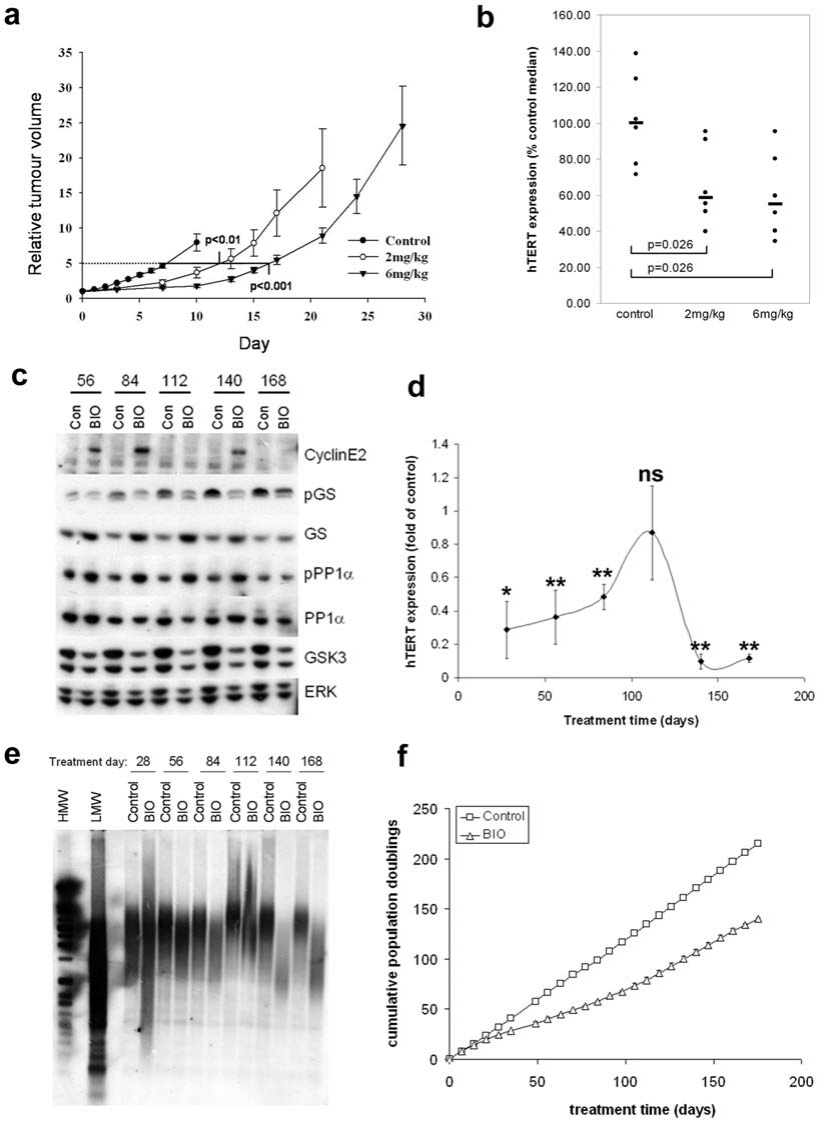
Therapeutic assessment of GSK inhibition. (A) Growth delay of A2780 xenografts. Athymic mice (n = 6/group) with established A2780 xenografts were treated intraperitoneally with BIO (2 mg/kg every two days, or 6 mg/kg twice weekly on days 1 and 4). Mean±SEM of calliper estimated tumour volumes relative to treatment day 0. (B) *hTERT* expression in A2780 xenografts. Tumours from vehicle or treated animals were excised and Q-RTPCR was performed. *hTERT* expression normalised to *RPS15* in each tumour is shown. Bars show median expression (control = 100%). (C) GSK3 inhibition during prolonged BIO treatment. A2780 cells were treated twice weekly on days 1 and 4 with 2.5 µM BIO or DMSO for 25 weeks. Analysis samples were taken every 4 weeks on treatment day 1. 20 µg protein samples were analysed by western blotting. Two experiments were analysed. Representative blots are shown. (D) Dynamic oscillation of *hTERT* expression. Control and treated samples from each time point were analysed by Q-RTPCR for *hTERT* expression normalised to *RPS15*. Mean±SEM of *hTERT* expression relative to control from two experiments (ns: not significant; *: p<0.5; **: p<0.01). (E) Prolonged BIO treatment shortens telomeres. 1 µg genomic DNA from control or treated cells was digested with HinfI/RsaI and southern blotted with DIG-labelled telomere sequence probe. Two independent treatments were assessed. Representative blots are shown. (F) Growth of A2780 cells during prolonged BIO treatment. Cells were counted weekly to determine cumulative population doublings (PD). Mean±SEM of two experiments.

To determine whether GSK3 inhibition suppresses *hTERT* expression in a tumour model, we inoculated A2780 into athymic mice. BIO (2 mg/kg every second day or 6 mg/kg twice weekly) or vehicle treatment was initiated via the intraperitoneal route when mean tumour diameters reached ∼0.5 cm. Tumours in control animals took 7.8 days to increase five-fold in volume, whereas the time taken was 13.1 days in the 2 mg/kg BIO group and 16.9 days in the 6 mg/kg BIO group (figure 6A). No overt toxicity was observed in treated groups. Therefore, BIO suppressed growth of established A2780 xenografts.

Animals were sacrificed after 3 weeks treatment at 2 mg/kg or 4 weeks treatment at 6 mg/kg. Tumours were harvested for QPCR analysis of *hTERT* expression (figure 6B). Median *hTERT* expression of the control group is fixed at 100% (range 71.82%–138.74%). BIO treatment suppressed *hTERT* expression to similar levels in both treatment groups. Median expression in the 2 mg/kg group was 58.62% that of the control group (range 39.85%–95.55%) and 55.01% in the 6 mg/kg group (40.5%–95.33%). The relative decrease in each treated group was statistically significant as determined by the Mann-Whitney test (p = 0.026). Therefore, GSK3 inhibition suppresses *hTERT* expression in established A2780 xenografts.

### Dynamic regulation of *hTERT* expression

To determine whether persistent inhibition of GSK3 results in telomere dependent growth arrest in cancer cells, A2780 were cultured in the presence of DMSO or 2.5 µM BIO for 25 weeks with twice weekly dosing as in the schedule in figure 3. Cells were counted weekly and analysis samples taken every four weeks. We again examined markers of GSK3 inhibition by western blotting (figure 6C). Because of decreasing growth rates, insufficient protein was obtained in day 28 BIO treated cells. However, in earlier experiments GSK3 was inhibited at this time point (figure 3D). As previously observed, GS phosphorylation was suppressed, its expression was increased and PP1α phosphorylation was also elevated until day 140. However, on day 168, little differential was observed between control and treated cells for GS expression or PP1α phosphorylation.

To extend this observation we assessed levels of cyclin E2 which increased after a single BIO dose (figure 3A). Cyclin E2 was increased in treated cells on days 56, 84 and 140, but not days 112 or 168. Thus, cyclin E2 apparently oscillated in treated cells. In contrast, GSK3 expression was reduced in treated cells at all time points as in the earlier time course. Therefore, persistent exposure to BIO had differential dynamic effects on downstream pathways.

To determine whether *hTERT* expression was suppressed throughout the time course, we performed RT-QPCR (figure 6D). Consistent with previous results, *hTERT* expression was strongly repressed on day 28 in BIO treated cells (29% of control). However, at subsequent time points until day 112, *hTERT* suppression was less efficient. On day 112, coinciding with the first loss of cyclin E2 induction, *hTERT* expression in treated cells was not significantly different from controls, as observed for 5637 cells on treatment day 35. Thereafter, expression decreased to a new low value on day 140 (10% of control) and remained at 12% of control on day 168. Thus, *hTERT* also appeared to oscillate in treated cells.

TRF analysis showed that a substantial lag phase preceded profound telomere shortening between days 112 and 140 in treated A2780. Densitometric estimates of average length on day 140 were ∼2 kb in treated cells, compared with ∼5 kb in control cells (Figure 6E). Therefore, persistent GSK3 inhibition significantly reduced telomere length of ovarian cancer cells. However, the blots also suggested that some extension occurred at days 112 and 168, which may be explained by the oscillation in *hTERT* expression and/or other effects on telomerase mediated by dynamic regulation of GSK3 effectors.

In these experiments, A2780 proliferation rapidly declined in the first weeks of treatment, though full culture crisis was not observed. While control cells grew steadily over the entire time course at ∼8.6 PD/week, growth of BIO treated cells decreased from 8.2 PD/week at day 7 to 3.5 PD/week at day 35, causing a partial growth plateau (figure 6F). Treated cells then grew at 3.5 PD/week until day 56, at which time growth accelerated, reaching a rate of ∼7 PD/week at day 126 which persisted for the rest of the time course.

### Network topology and dynamic behaviour

Although network modelling can successfully identify many of the players in a pathway, predicting the outcome of manipulating individual highly connected components of complex networks such as figure 5B is a challenge. However, genetic networks are mainly composed of recurring “wiring patterns” (network motifs) which can be modelled or even synthetically constructed to investigate relationships between motif topology and function [21]. Thus, identification of a network's constituent motifs may help to predict its behaviour. To better understand *hTERT* regulation by GSK3, we searched for several previously described motif types in the network model using MetaCore [21]–[23].

**Figure 7:**
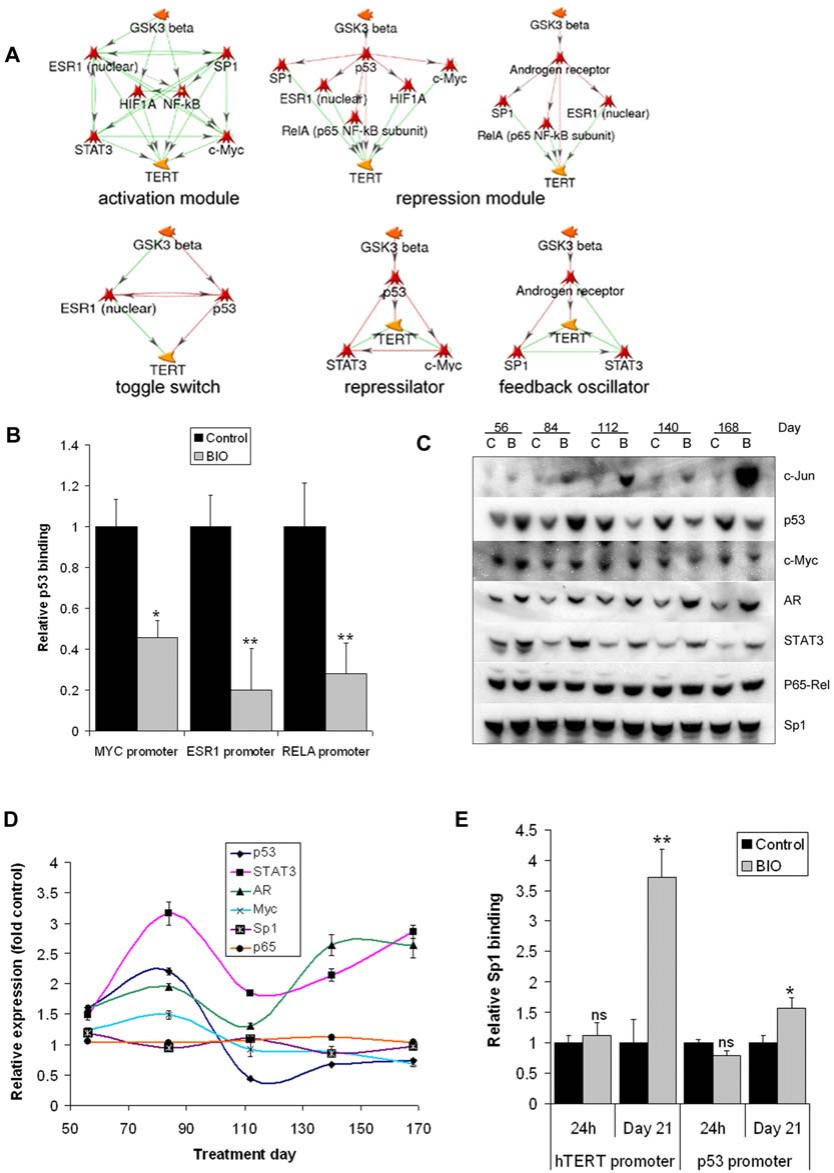
Network topology and dynamic behaviour. (A) Network motif analysis. Motifs in figure 5(B) were identified in MetaCore. Representative examples are shown. Green arrows: positive regulation; red arrows: negative regulation (reaction mechanisms not shown). (B) BIO regulates the repression module. A2780 were treated for 16 h with DMSO or 5 µM BIO prior to ChIP with p53 antibody and QPCR detection of indicated promoters. Mean±SEM of three experiments (*: p<0.05; **: p<0.01). (C) Dynamic regulation of network transcription factor expression levels under long term BIO treatment. Expression of network transcription factors in 20 µg protein samples from each time point of the 25 week time course were analysed by western blotting (C, Control; B, BIO treatment). Two independent treatments were analysed. Representative blots are shown. (D) Densitometry of (C): expression relative to control (c-Jun not shown due to scale). Mean±SEM of three measurements of each band. (E) Dynamic regulation of *hTERT* and *TP53* promoters by Sp1. A2780 were treated for 16 h with 5 µM BIO or 21 days with 2.5 µM prior to ChIP with Sp1 antibody and QPCR detection of the *hTERT* and *TP53* promoters. Mean±SEM of three experiments (ns: not significant; *: p<0.5; **: p<0.01).

We identified multiple potential reciprocal repression (toggle switch) and feedback oscillator motifs as in the examples in figure 7A, which may provide substantial scope for dynamic network behaviour. We also identified several types of coherent feed forward motifs. In particular, three distinct sub-networks form candidate activation and repression “modules”. The activation module links all positive regulators of *hTERT* via densely overlapping coherent type 1 motifs, while the repression module comprises several coherent type 2 motifs organised by p53 and AR which inhibit *hTERT* and its activators. Both motif types are reported to reduce noise in gene expression networks [21].

To further investigate the network structure, we determined the presence of the *MYC*, *RELA*, and *ESR1* gene promoters in the p53 ChIP experiments from figure 5D in which p53 binding to the *hTERT* promoter was decreased. We found that 16 h BIO treatment also reduced immunoreactive p53 at the *MYC* promoter by 2.2-fold, by 3.6-fold at *RELA* and 5-fold at *ESR1* (Figure 7B). Therefore, the early outcome of BIO treatment with respect to p53 includes co-ordinate regulation of the *hTERT* promoter and the promoters of at least three of its activators also present in the putative repression module sub-network.

Based on the proposed network topology, we hypothesised that prolonged GSK3 inhibition might affect dynamic behaviour of some transcription factors. Re-analysis of the 25 week time course revealed complex, fluctuating expression patterns for c-Jun, p53, c-Myc, AR and STAT3 expression, but not Sp1 or p65 in BIO treated samples (figures 7C and D). AR, STAT3 and c-Jun each showed a clear oscillation, particularly striking in the case of c-Jun, with a trough occurring on day 112 for AR and STAT3 and on day 140 for c-Jun. Levels of p53 were induced by BIO on days 56 and 84 but were lower than control at all other times. A similar, though less pronounced result was observed for c-Myc. Therefore, at least 5 transcription factors known to affect *hTERT* are subject to dynamic regulation under persistent GSK3 inhibition.

Since Sp1 levels were unaffected, we assessed whether its activity might be dynamically affected under prolonged GSK3 inhibition by ChIP in A2780 after 21 day 2.5 µM BIO treatments. Recovery of both *hTERT* and *TP53* promoters were analysed and compared with recovery after 16 h treatments. In contrast with the results after 16 h, immunoreactive Sp1 levels at the *hTERT* promoter were increased by 3.7-fold after 21 days treatment. Furthermore, Sp1 epitope at the *TP53* promoter was also increased by 1.6-fold at day 21. These data confirm that Sp1 is also dynamically regulated and participates in ongoing remodelling of both *hTERT* and *TP53* promoters under persistent GSK3 inhibition.

### Whole kinome siRNA screen of the *hTERT* promoter

Finally, to enable rational prediction of *hTERT* regulatory pathways overlapping with GSK3, we performed a whole kinome siRNA screen using the *hTERT* reporter in A2780. 3 independent siRNA against each of 719 kinase and kinase-related genes were assessed. Hit criterion was >2-fold change in promoter activity by at least 2/3 siRNA. A complex network of kinases controls activity of the transfected *hTERT* promoter, with 235/719 target genes in total scoring as hits. 232 were activators of the promoter (repression by siRNA) and only 3 were repressors.

**Figure 8:**
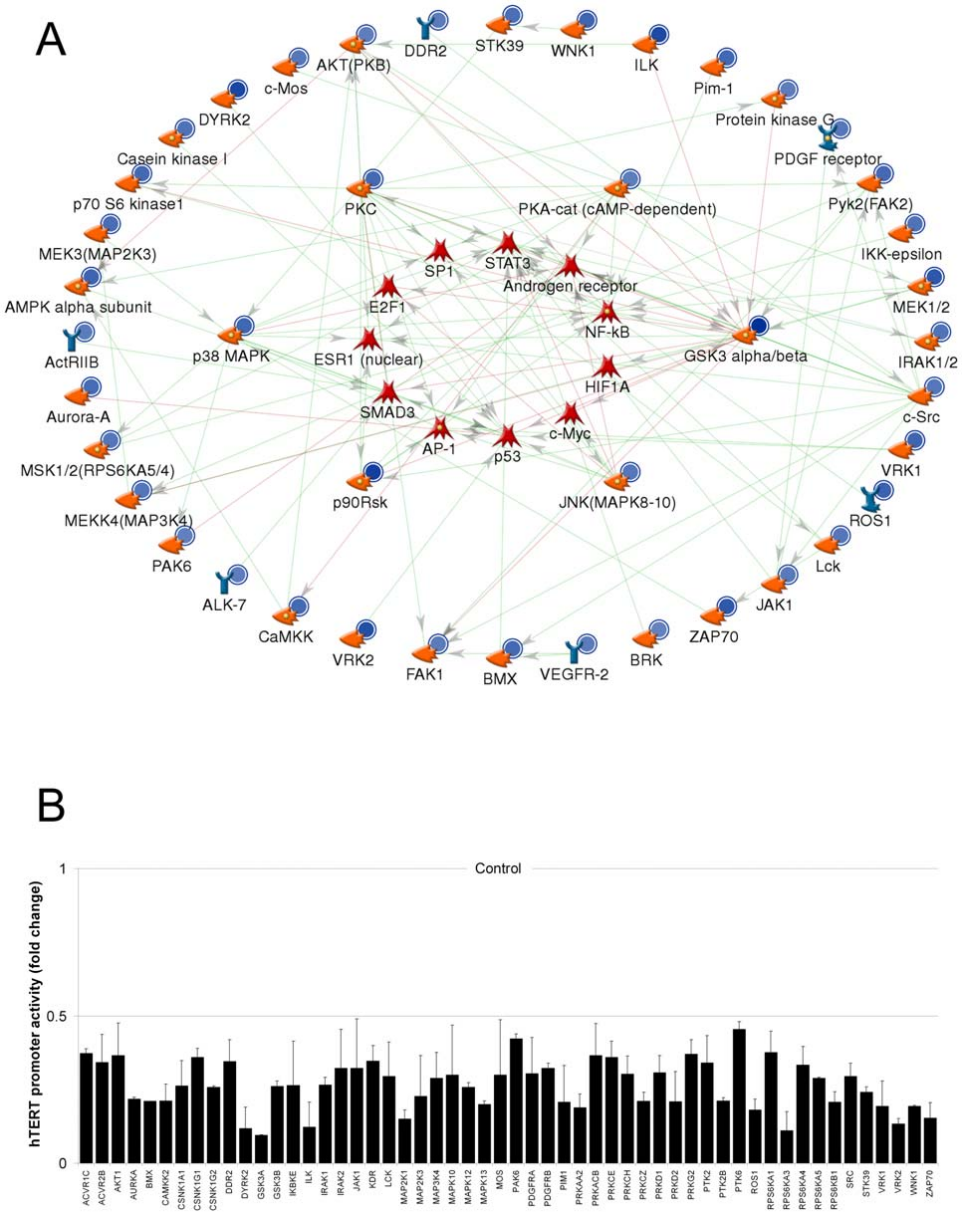
Whole-kinome RNAi screen of the *hTERT* promoter in A2780. (A) *hTERT*-luciferase was cotransfected with 50 nM siRNA in triplicate. 48 h post-transfection, luciferase assays were performed. 3 independent siRNA per target were assessed. Hit criterion was >2-fold change in promoter activity by at least 2/3 siRNA. 235 hit IDs were analysed in MetaCore using the “direct interactions” algorithm limited to phosphorylation interactions. Green arrows: positive regulation; red: negative regulation. Blue circles: siRNA repressed the *hTERT* promoter. Circle shading intensity indicates fold change (minimum 2-fold). Average derived from all independent hit siRNA is shown. (B) Relative repression of the hTERT promoter by siRNA against targets in the network model. Luciferase activities were calculated relative to control (non-specific) for each siRNA. Figure shows mean±SEM derived from all independent hit siRNA for each target.

We searched MetaCore for direct phosphorylation interactions between hits and network transcription factors (figure 8A; blue circles represent mean fold promoter repression with hTERT promoter activity values for each network target given in figure 8B). At least 54 hit kinases participate in upstream pathways and 38 hits directly phosphorylate one or more of the transcription factors. Critical divergence hubs with respect to the network transcription factors are GSK3 itself, p90-RSK, several PKC isoforms, PKA, JNK and p38. These hubs are predicted to be important modifiers of *hTERT* suppression with GSK3 inhibitors and preferred targets for combinatorial inhibition.

## Discussion

In this study we show that GSK3 activates *hTERT* gene expression and regulates telomere length homeostasis. We identified GSK3 as a potential pharmacological target to inhibit *hTERT* expression in a promoter screen of well defined kinase inhibitors. The result was confirmed using independent selective inhibitors of different chemotypes. BIO, GSK3β-specific siRNA, and genetic agonists of Wnt signalling all suppressed *hTERT* promoter activity in five cancer cell lines. Interestingly, over-expression of β-catenin increased promoter activity, suggesting a possible dual effect of Wnts on the *hTERT* promoter mediated by both canonical and non-canonical pathways. Regulation of endogenous *hTERT* by Wnts was not assessed in this study, though Wnt5A was previously shown to suppress telomerase in renal carcinoma cells [24]. Telomerase suppression by GSK3 inhibitors may partly involve Wnt pathways, but presumably also involves other GSK3 activities.

Multiplex phospho-specific western analysis in BIO treated A2780 revealed a complex functional signature involving altered expression of β-catenin, cyclins D1, B1 and E2, GS, as well as p90-RSK, STAT3 and IkB. Additionally, altered phosphorylation of AKT, p90-RSK, PP1α, cdc2 and STAT3 were observed. Observation of several markers over five weeks continuous treatment confirmed that GSK3 could be inhibited over prolonged periods.

Most importantly, BIO suppressed *hTERT* expression and telomerase activity with resultant telomere shortening over five weeks treatment in three cancer cell lines, indicating that a promoter screening approach can identify *bona fide* telomerase inhibitors. Microarray and network analysis of GSK3 inhibited A2780 suggested that the activities of multiple transcription factors could be altered. We defined a candidate network linking GSK3 and *hTERT* via some or all of Sp1, E2F1, STAT3, SMAD3, ESR1, AR, HIF-1α, NFκb, AP-1, p53 and c-Myc.

In support of the network model, 16 h BIO treatment altered binding affinity of A2780 nuclear extracts to multiple consensus oligonucleotides, including sites for c-Myc, NFκB, SMAD3, p53, Sp1 and STAT3. Critically, ChIP analysis indicated that immunoreactive c-Myc, p53 and NFκB p65 were reduced at the endogenous *hTERT* promoter by 16 h BIO treatment while STAT3 was increased. It should be noted that ChIP results may reflect changes either in DNA binding or in epitope masking. In either case, GSK3 inhibition causes rapid and widespread remodelling of the *hTERT* promoter involving at least these factors and possibly others suggested by the network and oligo binding analyses. Both activators and repressors of *hTERT* were affected, underscoring the difficulty of interpreting the transcriptional effect in terms of single factors.

GSK3 regulates several transcription factors directly and effectors PTEN, β-catenin, p21, FAK1, Tuberin, cyclin D1 and cyclin D2 may also play a role. We have not directly addressed their roles in this study, though FAK1 siRNA suppressed the *hTERT* promoter in A2780 and β-catenin over-expression up-regulated the promoter in several cell lines. Furthermore, BIO increased expression of β-catenin and cyclin D1 in A2780 cells and reduced AKT phosphorylation which may occur downstream of PTEN [25]. Previous studies have shown regulation of *hTERT* and/or telomerase in various experimental settings by several of these factors [26], [27].

Consequently, GSK3 may control *hTERT* expression in a broad range of cells, including those with mutation or disruption in one or more branches of the network as in 5637 (mutant p53) or HCT116 (mutant β-catenin). Thus, GSK3 could be an attractive pharmacological target for broad spectrum suppression of telomerase in cancer cells. In support of a therapeutic application, BIO caused tumour growth delay and inhibited endogenous *hTERT* expression in established A2780 xenografts without overt toxicity. Unexpectedly, however, prolonged inhibition of GSK3 in cultured A2780 did not lead to stable *hTERT* suppression, although telomere lengths were profoundly reduced. Rather, dynamic oscillation of *hTERT* was observed.

Complex inter-transcription factor interactions are expected from the model. Upstream of *hTERT* are multiple densely overlapping coherent type 1 feed-forward motifs mainly organised by ESR1 and Sp1 (activation module). A series of coherent type 2 motifs emanates from AR and p53 (repression module). Both motif architectures may reduce the impact of transient noise in genetic networks and may therefore govern stable activation or repression of *hTERT* [21]. Although direct validation of individual motifs is beyond the scope of this study, BIO reduced p53 binding at *hTERT*, *MYC*, *RELA*, and *ESR1* promoters in ChIP experiments, suggesting coordinated functional regulation of transcriptional interactions consistent with the proposed topology of the repression module. The network also contains multiple potential switch and feedback oscillator motifs, suggesting there is substantial scope for dynamic network behaviour [21]–[23].

Indeed, several network components, including p53 and NFκB are known to exhibit dynamic oscillations under certain conditions [28], [29]. Inter-transcription factor interactions may provide another mechanism for dynamic behaviour. Notably, GSK3 directly controls stability of several transcription factors [30]. An interesting possibility is that GSK3 exerts a “compressor” effect which fine-tunes the overall network steady state. Consistent with this interpretation, expression of several transcription factors varied dynamically over long term GSK3 inhibition.

Currently, the frequency of these fluctuations is unknown and may even involve stochastic events. Therefore, other apparently unaffected factors may also be regulated at different time points, or their activities may be subject to dynamic changes independently of expression, as would appear to be the case with Sp1. Overnight BIO treatment had no effect on Sp1 levels at the *hTERT* promoter and its expression was unaffected in the time course. However, 3 week treatments did increase Sp1 epitope at both the *hTERT* and *TP53* promoters, confirming that it participates in dynamic regulation of *hTERT* and other network components.

Knowledge of other druggable target pathways affecting the network may provide strategies to complement and/or stabilise telomerase inhibition. To discover other candidate kinase targets potentially affecting *hTERT*, we performed a whole kinome siRNA screen using the *hTERT* reporter, revealing 235 kinase genes that regulate promoter activity in A2780, of which at least 54 phosphorylate components of the network model. Key hubs were GSK3 itself, p90-RSK, several PKC isoforms, PKA, and the JNK and p38 MAP kinases. Future studies will determine their functional involvement in regulation of telomerase by GSK3.

In summary, our results suggest computational and screening approaches combined with appropriate focused validation efforts may lead to a more nuanced understanding of telomerase gene regulation. Our data lend support to the emerging prospect of GSK3 inhibitor therapy of cancer, both from the standpoint of telomerase inhibition and because of the rapid xenograft growth reduction effect observed which may also involve additional effects of GSK3 inhibition [31]. However, prolonged GSK3 inhibition has complex network effects, at least in cancer cells. Therefore, combinatorial regimens may be most appropriate. This finding may also have implications for the use of single agent GSK3 inhibitors in the settings of bipolar disorder, Alzheimer's disease and diabetes in which long term treatment schedules are also required. We have identified several markers of dynamic network behaviour. Their examination in suitable model systems for these disorders may also be advisable. Finally, GSK3 inhibition has been proposed as a method to expand stem and progenitor cell pools. Protocols involving telomerase positive progenitors should also address telomere status.

## Materials and Methods

### Cell lines, plasmids, siRNA and inhibitors

Cells used were: 5637 bladder carcinoma, C33A cervical carcinoma, A549 lung adenocarcinoma, and HCT116 colon carcinoma cells, obtained from ATCC, and A2780 ovarian adenocarcinoma cells, originally obtained from Dr RF Ozols [32]. Reporter pGL3-*hTERT* contains the *hTERT* promoter region -585/-9, relative to the translational start site. Plasmids pCMV-mWnt3 and pCMV-mWnt5a were kindly provided by Dr. Mejlinda Lako (Institute for Ageing and Health, Newcastle University, UK). Human DVL2 and β-catenin expression vectors were obtained from Origene (Rockville, MD). The whole kinome siRNA library, non-specific siRNA and GSK3β-specific siRNA (siRNA 42839: sense sequence 5′-GGACAAGAGAUUUAAGAAUtt; siRNA 203: sense sequence 5′-GGUGACAACAGUGGUGGCAtt) were obtained from Applied Biosystems (Warrington, UK). The kinase inhibitor library was obtained from Biomol International Ltd (UK). BIO, AR-A014418, TWS119, Roscovitine, and 1-MeBIO were obtained from EMD Biosciences (Nottingham, UK).

### Transfections and luciferase assay

All transfections were performed in quadruplicate using lipofectamine according to the manufacturer's instructions using a 2∶1 ratio reagent:DNA (Invitrogen, Paisley, UK). Under these conditions, transfection efficiencies were found by pSV40-βGal assay to be: HCT116, 51%; A549, 39%; A2780, 27%; 5637, 29%; C33A, 28%. 250 ng *hTERT* reporter plasmid per well was transfected in 96-well luminometer plates (Fisher Scientific UK, Leicestershire, UK). 32 h post-transfection cells were exposed to inhibitors for 16 h. In cotransfections, 250 ng expression vectors or 50 nM siRNAs were included. 30 ng pSV40-Renilla luciferase expression plasmid (Promega Ltd, Madison, WI) was included in each well for normalisation. 48 h post-transfection, luciferase activities were determined using dual luciferase assay reagents according to the manufacturer's instructions (Promega Ltd, Madison, WI). All experiments were repeated at least 3 times.

### MTT assay

Cells were seeded in quadruplicate wells and triplicate 96-well plates 2 days prior to addition of inhibitor titrations. Cells were exposed to inhibitors for 16 h then incubated for an additional 3–4 days prior to MTT assay (MTT supplied by Sigma (Dorset, UK)). MTT reduction assays were performed using Softmax Pro 4.6 software (Molecular Devices Ltd., Wokingham, UK). All experiments were repeated at least 3 times.

### Western blotting

Protein extracts were prepared in passive lysis buffer (Promega Ltd, Madison, WI). Protein concentrations were estimated at OD595 using the BioRad protein assay (BioRad Laboratories Ltd, Hemel Hempstead, UK). 20 µg protein for singleplex experiments, or 1 mg protein for multiplex analysis, were separated by SDS-PAGE, blotted onto PVDF filter (Millipore, Watford, UK) and blocked overnight in PBS-T containing 5% non-fat dried milk. All antibodies are listed in supporting file S1. For multiplex analysis, membrane was separated into lanes using Immunetics' miniblotter 28 dual apparatus (Web Scientific, UK). Primary antibodies were detected with HRP-conjugated secondary. HRP was detected using ECL HRP detection reagents (Amersham Pharmacia, Buckinghamshire, U.K.). All experiments were performed at least twice.

### ChIP assays

DMSO or BIO treated cells were harvested at 70%–80% confluence. ChIP assays were performed following instructions of the kit supplier (Millipore, Watford, UK). Cell layers were fixed in formaldehyde and lysed in SDS buffer with protease inhibitors. Chromatin fragments of 500 bp-1 kb were generated by sonication using a Branson S25OD sonifier (Branson Ultrasonics Corp., Danbury, CT). All antibodies are listed in supporting file S1. Each assay included a no antibody control. Each promoter was detected by Q-PCR in triplicate using Genetic Research Instrumentation Opticon monitor equipment and software (Essex, UK) and sybr green fluorophore. Promoter-specific primers used were: *hTERT*, 5′-CATTCGTGGTGCCCGGAGC and 5′-GCCCCAGCGGAGAGAGGTCG or 5′-GCGACCTGTAATCCTAAGTATT and 5′-GGGTTGCTCAAGTTTGGATCTAA for p53 binding analysis; *TP53*, 5′-GCACCAGGTCGGCGAGAATCCTG and 5′-CGTGGAAAGCACGCTCCCAGCC; *ESR1*, 5′- CCAATGTCAGGGCAAGGCAA and 5′-GGAGCCTGCGGGTCCGGTGAA; *RELA*, 5′-AGTTCAACCACCCGGCCTCT and 5′-GAGGGTGGGTCCGCCGATTA; *MYC*, 5′-GCTGCCCGGCTGAGTCTCCTCCC and 5′-CCTCCCCACCTTCCCCACCCTC. Optical read temperatures were optimised to exclude primer dimers. All ChIP experiments were performed at least 3 times. Q-PCR was repeated twice for each experiment.

### TRAP assay

The TRAPeze XL kit was used for TRAP assay according to the manufacturer's instructions (Millipore, Watford, UK). Cell pellets were lysed in CHAPS lysis buffer and protein concentrations estimated by Bio-Rad assay (BioRad Laboratories Ltd, Hemel Hempstead, UK). 0.5 µg protein was mixed with TRAPeze reaction mix containing TS primer, fluorescein labelled RP primer, control template and sulforhodamine labelled control K2 primer. Each assay included no-telomerase, no-Taq, and heat-treated controls. Extension products were generated at 30C followed by Q-PCR detection in triplicate using Chromo4 equipment and software (BioRad Laboratories Ltd, Hemel Hempstead, UK). Total product generated was measured against TR8 standards and normalised to the ROX internal control. All experiments were performed three times and the TRAP assay was repeated twice for each experiment.

### TRF analysis

Telomere length assays were performed using the teloTAGGG kit according to the manufacturer's instructions (Roche Diagnostics Ltd., West Sussex, UK). 1 µg genomic DNA from cell pellets was digested with HinfI/RsaI. Digestion products were separated by gel electrophoresis alongside DIG-labelled molecular weight markers and blotted onto positively charged nylon membrane (Roche Diagnostics Ltd., West Sussex, UK). Membranes were UV cross-linked, baked at 120C and washed in 2×SSC solution. Hybridisation of the DIG-labelled telomeric probe was performed using buffers and probe provided. Finally, membranes were washed, probed with alkaline phosphatase conjugated anti-DIG and exposed to the CDP-star substrate. All experiments were performed at least twice.

### Quantitative RT PCR

Q-PCR was performed in triplicate using Genetic Research Instrumentation (Essex, UK) Opticon monitor equipment and software. Sybr green was used as fluorophore. The primers used were: *RPS15*, 5′-TTCCGCAAGTTCACCTACC and 5′-CGGGCCGGCCATGCTTTACG; *hTERT* 5′-CTGCTGCGCACGTGGGAAGC and 5′-GGACACCTGGCGGAAGGAG. Optical read temperatures were optimised to exclude primer dimers. All treatments were repeated three times and Q-PCR was performed twice for each assay. Splice variant PCR was performed with primers 5′-GCCTGAGCTGTACTTTGTCAA and 5′-GCCAAACAGCTTGTTCTCCATGTC and analysed using an Agilent Bioanalyser 2100 and DNA-1000 assay chips (Agilent Technologies, Santa Clara, CA).

### Microarray processing

RNA from 3 independent treatments was labelled and amplified using the two-colour microarray gene expression analysis protocol (Agilent Technologies, Santa Clara, CA). Control cell RNA was labelled with cyanine 3-CTP and BIO treated cell RNA labelled with cyanine 5-CTP. 750 ng of cy-3 and cy-5 labelled, amplified cRNA were mixed and hybridised in duplicate to 44 k Agilent whole human genome microarrays, according to the manufacturers instructions and incubated for 17 hrs at 60°C in a rotating hybridisation oven. Arrays were washed on a magnetic stirrer using Agilent wash buffers. Slides were scanned on an Agilent DNA microarray scanner at 5 µm resolution, PMT at 100% and 10%. The extended dynamic range setting corrected for saturation.

### Microarray data analysis

Microarray data was processed in line with the Microarray Gene Expression Data Society (http://www.mged.org/) to standardize the presentation of microarray data. MIAME compliant data have been deposited for public access in the Gene Expression Omnibus (GEO) at http://www.ncbi.nlm.nih.gov/geo/ with the accession number GSE14532. Data was extracted using Agilent Feature Extraction software version 8.1 (Agilent Technologies). Background-subtracted data for separated red and green channels was imported into GeneSpring GX 7.3.1 (Agilent Technologies, Santa Clara, CA) for normalisation and statistical analysis. Intra-array normalisation was carried out using the 50^th^ percentile for each microarray. Significant differences in expression between DMSO control and BIO treated cells were determined using Welch analysis of variance (ANOVA) assuming normality, but not equal variances and Benjamini and Hochberg false discovery rate multiple testing correction of 5%. IDs with >5-fold intensity change, p<0.01 were selected for further analysis.

### MetaCore network analysis

Differentially expressed genes from supporting file S2 were analysed using the “transcription regulation” algorithm in MetaCore from GeneGo Inc. (filters: positive and negative interaction types; all mechanisms). All genes from the 10 most significant returned networks were merged to an enriched list and analysed using the “auto expand” algorithm (filters: positive and negative interaction types; all mechanisms). A best fit transcriptional network was identified by varying the network size. Edges linking high-degree transcription factors with GSK3 were identified using the “analyse network” algorithm (filters: positive and negative interaction types, all mechanisms). For kinome-wide RNAi analysis, edges linking hits with high-degree transcription factors from the candidate pathway were identified using the “direct interactions” algorithm (filters: positive and negative phosphorylation interaction types only, kinase and transcription factor object-types only). Algorithms are described in [20]. All interactions in MetaCore are manually compiled from full text articles. All references are available on request.

### Multiplex oligonucleotide binding assay

Consensus oligonucleotide binding assay was performed using the protein/DNA array I kit from Panomics (Freemont, CA) according to the manufacturers instructions. 10 µg nuclear extracts prepared with Panomics nuclear extraction kit were incubated with biotin labelled consensus oligonucleotide probe mix. Bound probes were isolated on spin columns, denatured and hybridised to nylon membrane containing a consensus transcription factor binding sequence array. Membranes were washed in hybridisation wash I and wash II then blocked using blocking buffer supplied. Finally, membranes were incubated with streptavidin-HRP, washed, and labelled probes were detected with chemiluminescent detection reagents supplied.

### Xenograft experiments

Animal studies were carried out under an appropriate United Kingdom Home Office Project Licence and all work conformed to UKCCR Guidelines for welfare of animals in experimental neoplasia. 10^7^ A2780 cells in PBS were injected subcutaneously into the right flank of CD1 *nu/nu* mice (Charles River). After 7 to 10 days when mean tumour diameter was ∼0.5 cm (day 0), animals were randomized in groups of 6. A stock solution of 2 mg/ml BIO was prepared and diluted into PBS immediately before injection. Mice were treated intraperitoneally with BIO at a dose of 2 mg/kg on alternate days or at a dose of 6 mg/kg twice weekly on days 1 and 4. Tumour volumes were estimated by calliper measurements assuming spherical geometry (volume = d^3^×π/6).

### Densitometry

Densitometry was performed on telomere length experiments, western blots and multiplex oligonucleotide binding experiments using a BioRad GS-800 densitometer (BioRad Laboratories Ltd, Hemel Hempstead, UK) and Quantity One software.

### Statistical analysis

Statistical analysis of all experiments was performed by one way ANOVA except *hTERT* expression in xenografts, which was analysed by Mann-Witney U test.

## Supporting Information

Figure S1 Chemical structures of GSK3 inhibitors reported in the study.
(0.14 MB TIF)

Figure S2 BIO, but not MeBIO, activates β-catenin signalling.
(0.07 MB TIF)

Figure S3 Regulation of Topflash reporter activity by over-expression of Wnt pathway components.
(0.15 MB TIF)

Figure S4 BIO selectively represses expression of the full length hTERT transcript in A2780.
(0.94 MB TIF)

Figure S5 Representative results of MetaCore “transcriptional-regulation” algorithm analysis.
(3.60 MB TIF)

Figure S6 Optimisation of the best-fit transcriptional network.
(1.23 MB TIF)

Figure S7 Expression of hTERT after 16 h treatment with 5 µM BIO
(1.92 MB TIF)

Supporting File S1 Antibodies used in the study.
(0.02 MB XLS)

Supporting File S2 Differentially expressed Agilent IDs and fold intensity change.
(0.09 MB XLS)

Supporting File S3 Best fit transcriptional network statistics.
(0.21 MB XLS)

Supporting File S4 Legends to supporting figures and files
(0.03 MB DOC)
