# Supplementary figures and images for: Dynamic Telomerase Gene Suppression via Network Effects of GSK3 Inhibition
Source: PLoS One. 2009 Jul 31;4(7):e6459. doi: 10.1371/journal.pone.0006459 (PMC2714081; doi:10.1371/journal.pone.0006459)

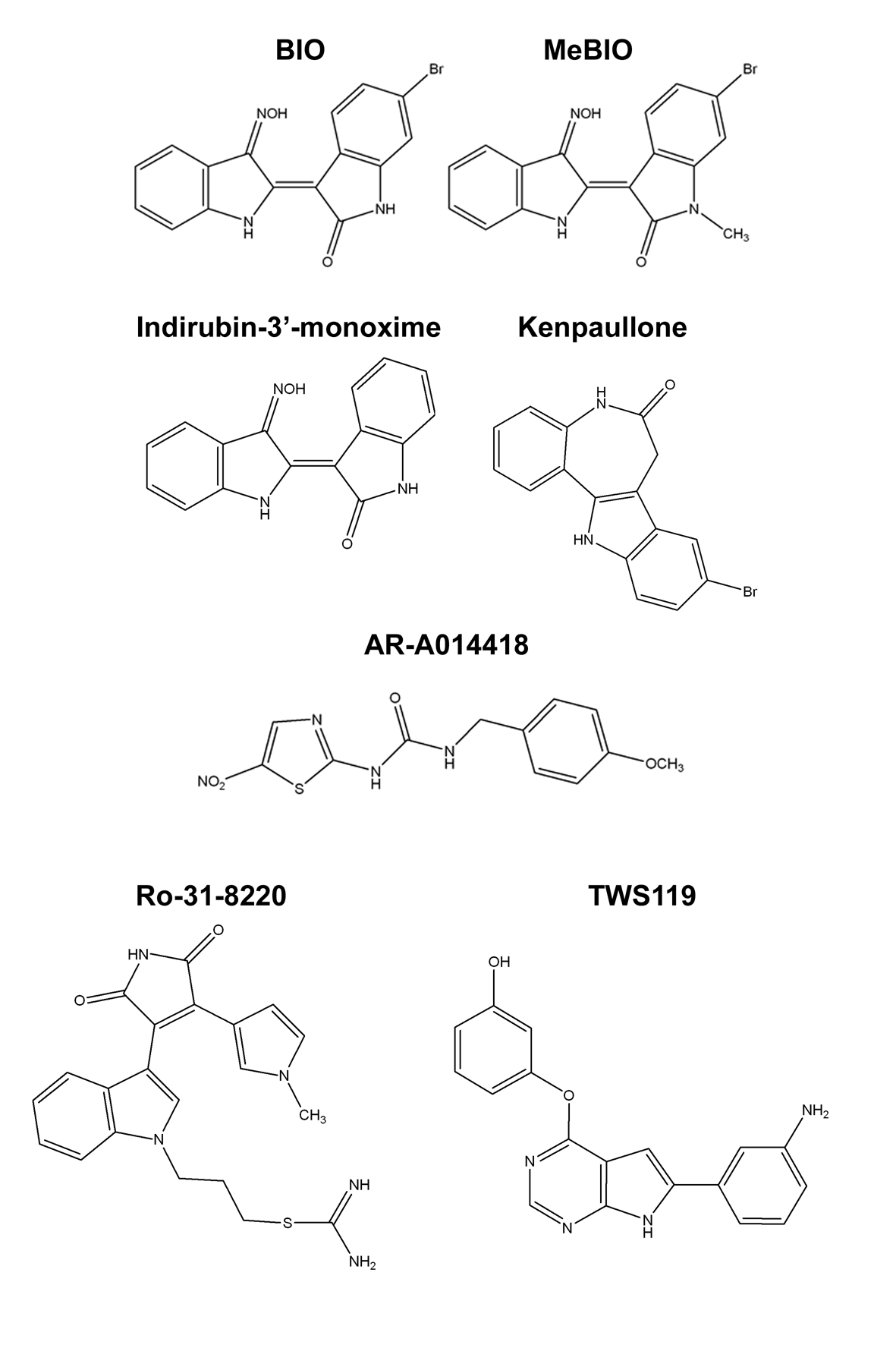

Supplement: Figure S1 — Chemical structures of GSK3 inhibitors reported in the study. (0.14 MB TIF) [file pone.0006459.s001.tif]

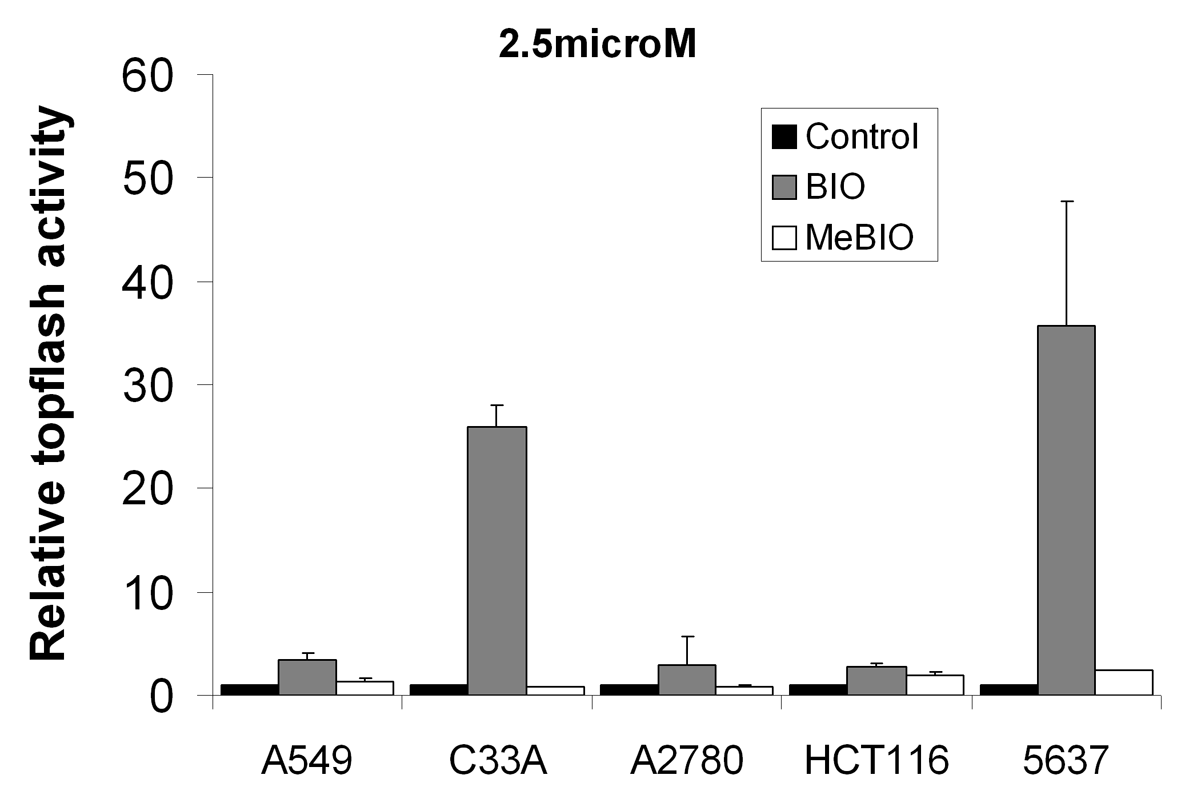

Supplement: Figure S2 — BIO, but not MeBIO, activates β-catenin signalling. (0.07 MB TIF) [file pone.0006459.s002.tif]

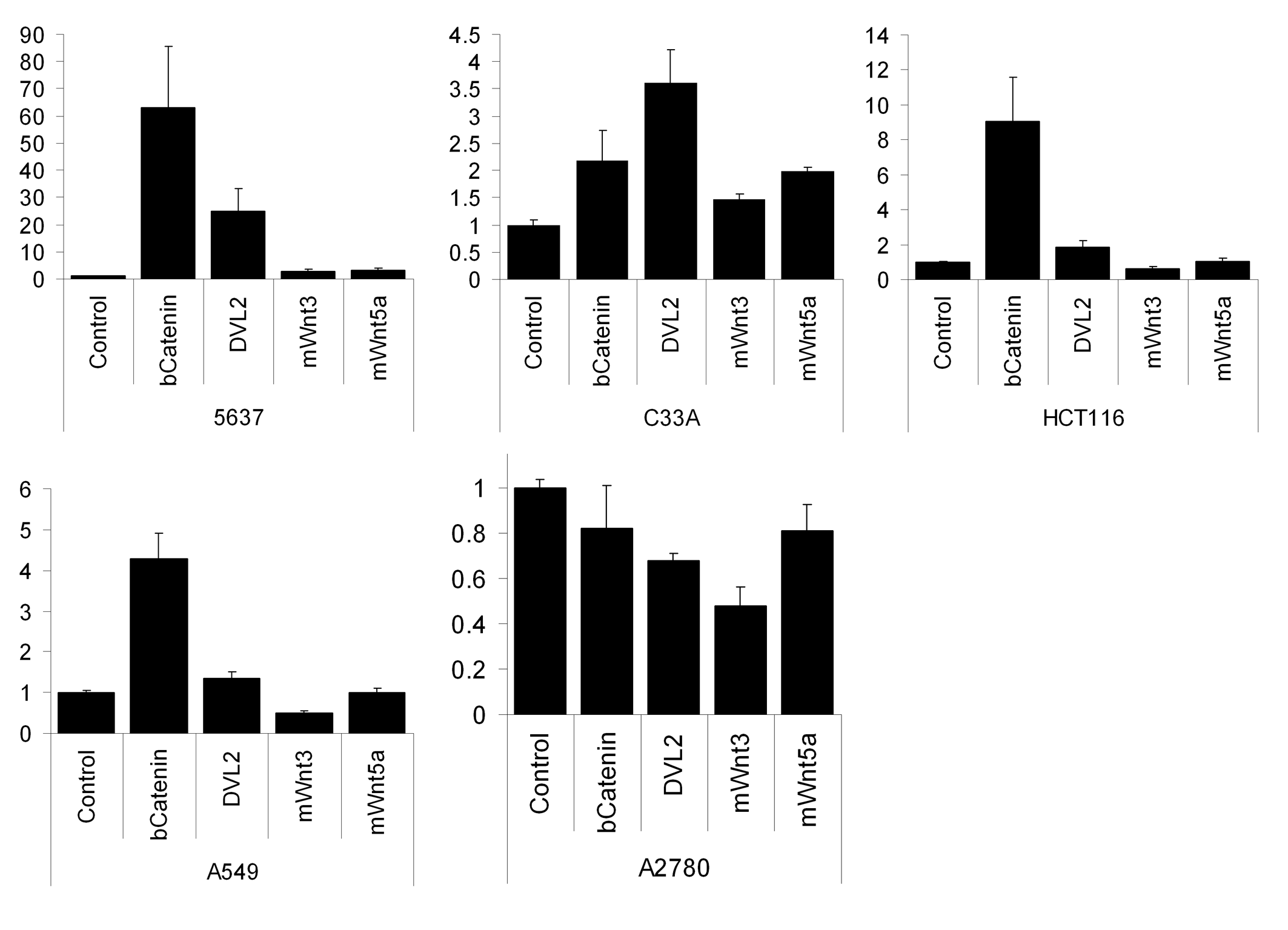

Supplement: Figure S3 — Regulation of Topflash reporter activity by over-expression of Wnt pathway components. (0.15 MB TIF) [file pone.0006459.s003.tif]

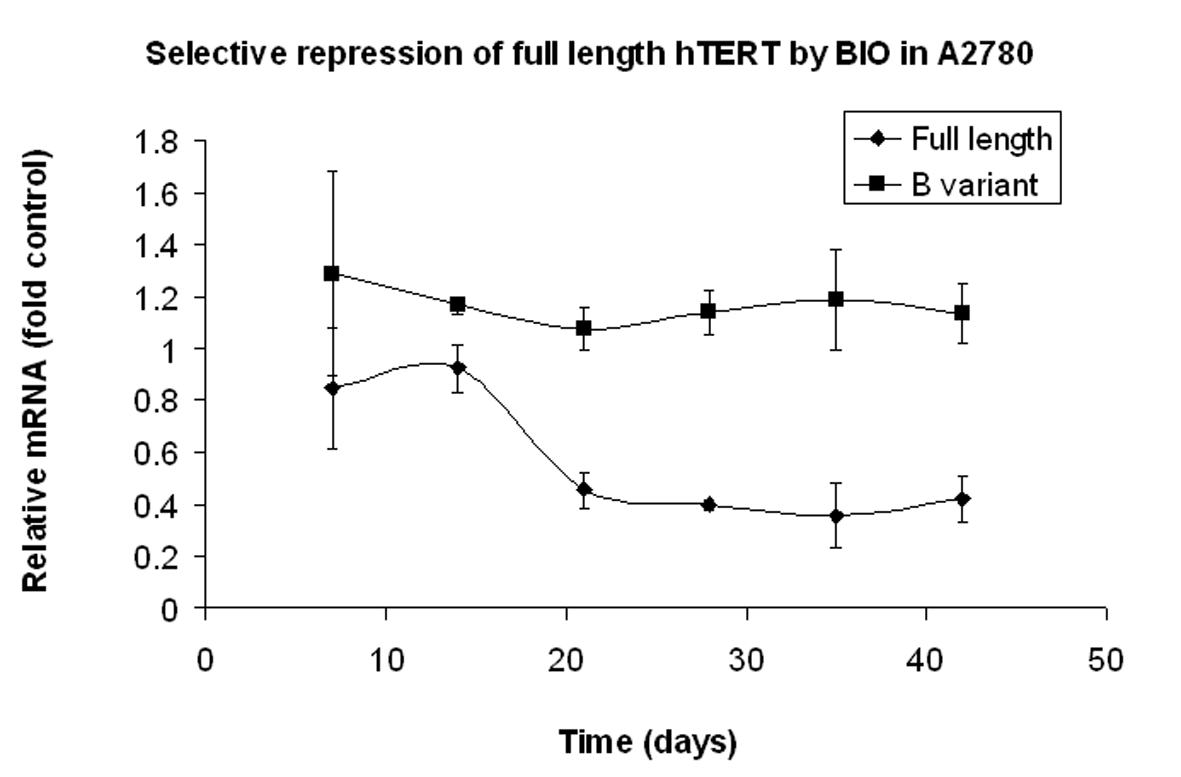

Supplement: Figure S4 — BIO selectively represses expression of the full length hTERT transcript in A2780. (0.94 MB TIF) [file pone.0006459.s004.tif]

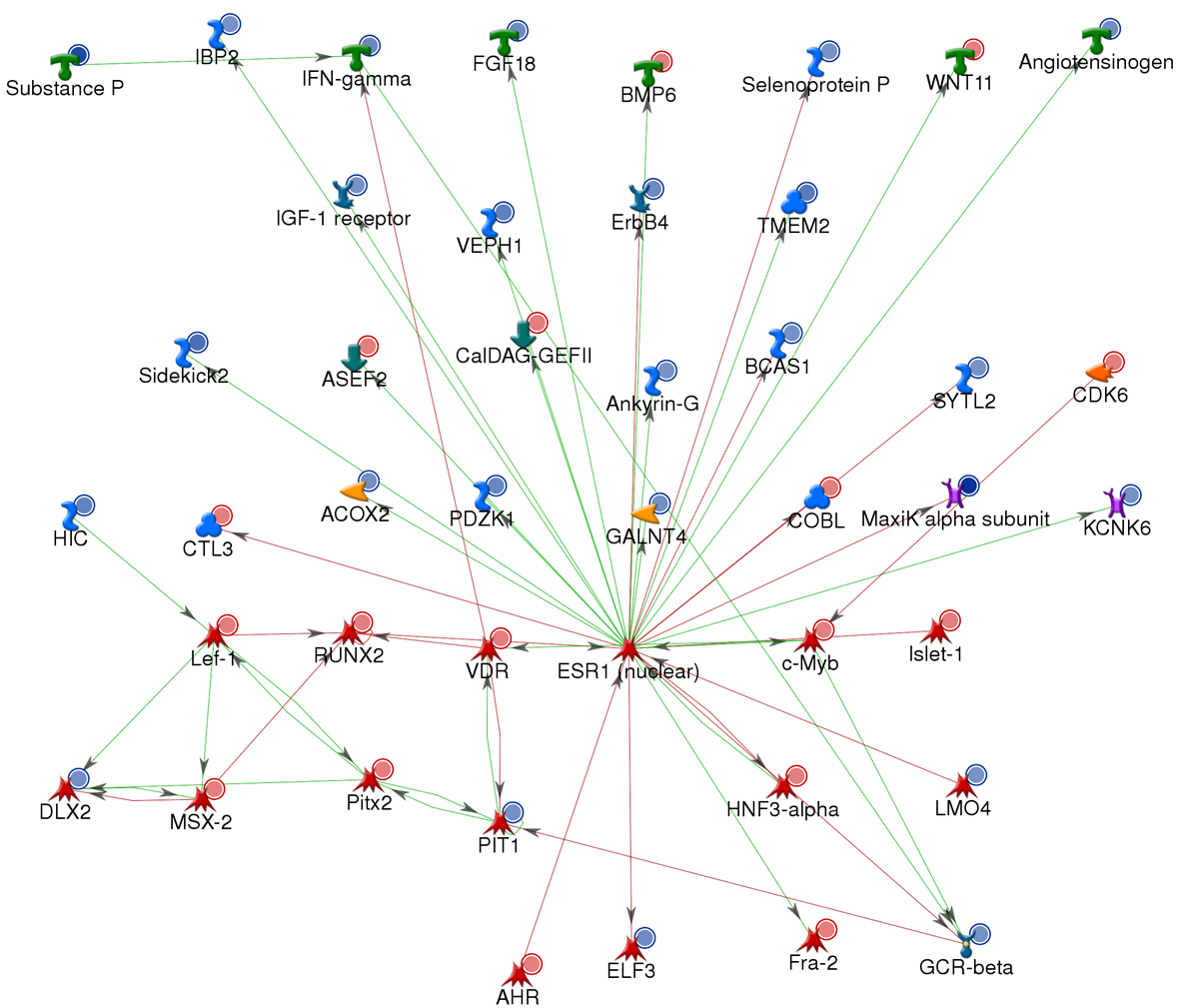

Supplement: Figure S5 — Representative results of MetaCore “transcriptional-regulation” algorithm analysis. (3.60 MB TIF) [file pone.0006459.s005.tif]

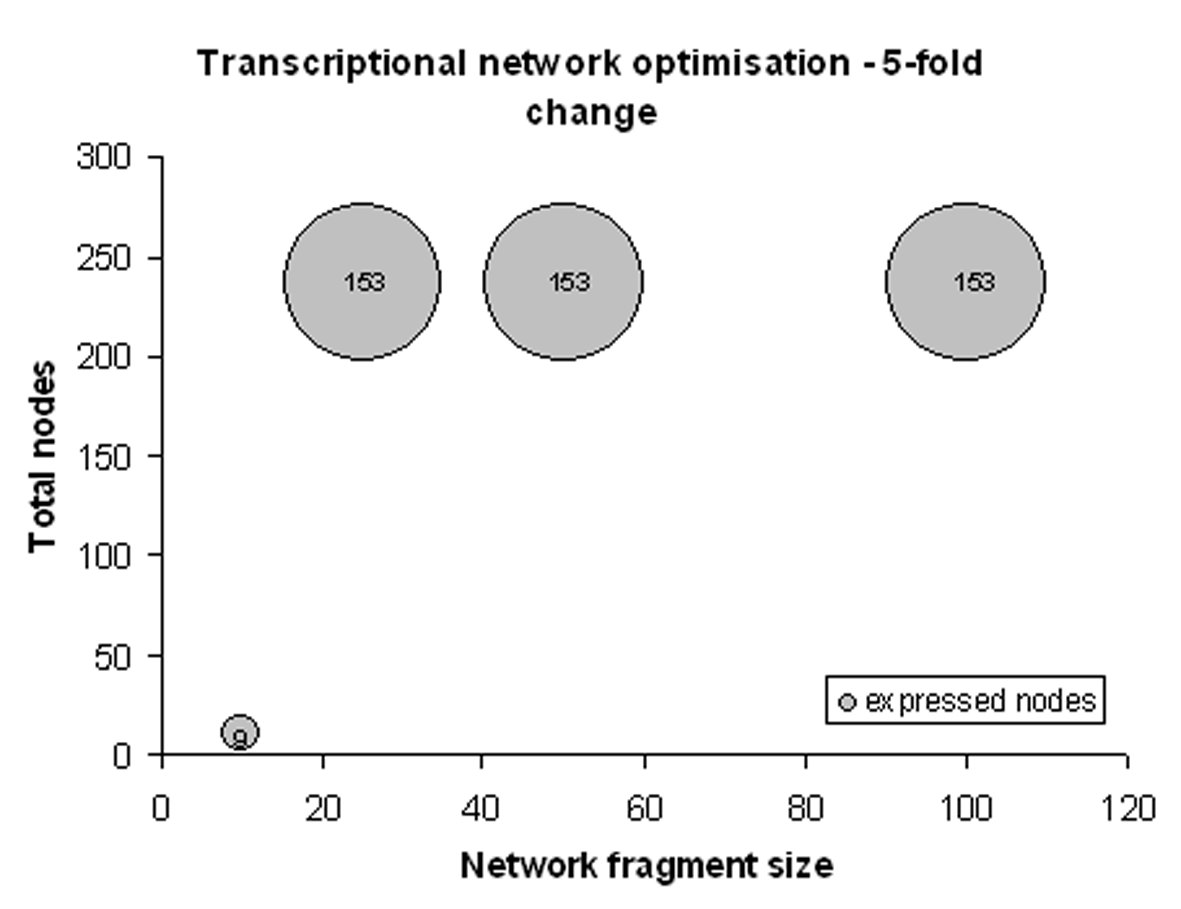

Supplement: Figure S6 — Optimisation of the best-fit transcriptional network. (1.23 MB TIF) [file pone.0006459.s006.tif]

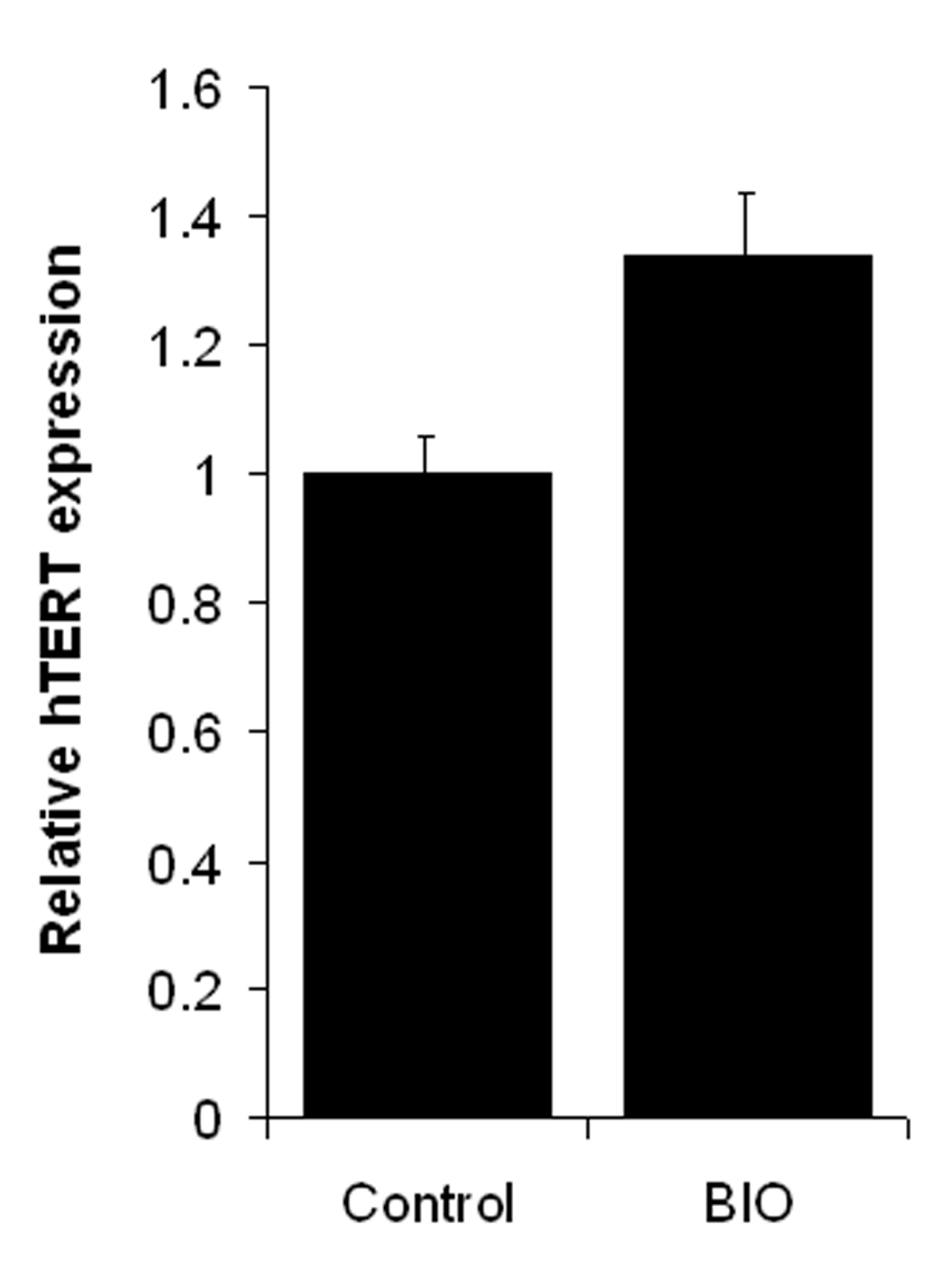

Supplement: Figure S7 — Expression of hTERT after 16 h treatment with 5 µM BIO (1.92 MB TIF) [file pone.0006459.s007.tif]
