# Supplementary material for: Dynamic Telomerase Gene Suppression via Network Effects of GSK3 Inhibition
Source: PLoS One. 2009 Jul 31;4(7):e6459. doi: 10.1371/journal.pone.0006459 (PMC2714081; doi:10.1371/journal.pone.0006459)
Supplement: Supporting File S4 — Legends to supporting figures and files (0.03 MB DOC) [file pone.0006459.s011.doc]

**Supporting figure S1**

Chemical structures of GSK3 inhibitors reported in the study.

**Supporting figure S2**

BIO, but not MeBIO, activates β-catenin signalling. Cell lines shown were transfected in triplicate with 250ng Topflash luciferase reporter. 32h later cells were treated for 16h with BIO or MeBIO at 2.5μM for 16h prior to luciferase assay. Mean ± SEM of 3 experiments.

**Supporting figure S3**

Regulation of Topflash reporter activity by over-expression of Wnt pathway components. Cell lines shown were transfected in triplicate with 250ng Topflash luciferase reporter and 250ng expression vectors indicated. 48h later luciferase assays were performed. Mean ± SEM of 2 experiments.

**Supporting figure S4**

BIO selectively represses expression of the full length *hTERT* transcript in A2780. Control and treated samples from each time point were analysed by Q-RTPCR for ribosomal S15 expression. 100ng/uL S15 equivalents were added to semi-quantitative PCR reactions and quantified using the Agilent Bioanalyser 2100 and DNA-1000 assay chips. Mean and standard errors from three independent 5 week treatments are shown. Expression of *hTERT* transcripts in BIO treated cells relative to controls is shown.

**Supporting figure S5**

Representative results of MetaCore “transcriptional-regulation” algorithm analysis. Differentially expressed genes were identified with Agilent whole genome expression arrays (n=3; mean fold change>5; p<0.01). Networks returned by transcription-regulation analysis centre on high-degree transcription factor neighbours of differentially expressed genes. Network for ESR1 is shown. Blue circles: genes downregulated in BIO treated cells; red circles: upregulated genes. Circle shading intensities indicate fold change (minimum 5-fold). Arrows represent biological effects (interaction mechanisms not shown). Green: activation; red: inhibition.

**Supporting figure S6**

Optimisation of the best-fit transcriptional network. MetaCore auto-expand algorithm was performed on 144 network object IDs exported to an enriched gene list. Prior to execution of the algorithm, maximum network size was adjusted by setting the nodes/fragment between 10 and 100 nodes/fragment using “advanced options”. The optimal network had 25 nodes/fragment and featured 237 nodes in total, of which 144 were differentially expressed.

**Supporting figure S7**

Expression of *hTERT* after 16h treatment with 5μM BIO. A2780 cells were treated with DMSO or BIO and subsequently harvested for Q-RTPCR for *hTERT* or ribosomal S15. Expression of *hTERT* was normalised to S15. Mean and standard error of *hTERT* expression relative to control from three independent experiments is shown.

**Descriptions of supporting files:**

**Supporting file S 1**

File contains a list of all primers, antibodies and suppliers used in the study.

**Supporting file S2**

1048 differentially expressed Agilent IDs with fold change in intensity in BIO treated cells relative to control.

**Supporting file S 3**

File contains detailed information about the best-fit transcriptional network, including network object expression levels and degrees. Options tab: network build options. Experiments tab: expressed genes present in network. Nodes tab: all objects present in network. Other tabs show hubs, transcription factors, membrane proteins and secreted proteins present in network. Edge numbers: green, total edges in network; red, hidden edges (present in network but not shown).
